# Assessment of microbial growth in various aligner materials

**DOI:** 10.6026/973206300221574

**Published:** 2026-03-31

**Authors:** T.G.R Nitharshini, R Saravanan, M.K Karthikeyan, N Raj Vikram, Raja Kumar Raja Kumar, Jeevana Poovalingam

**Affiliations:** 1 Department of Orthodontics and Dentofacial Orthopaedics, Thai Moogambigai Dental College and Hospital, Dr. MGR Educational and Research Institute, Chennai, Tamil Nadu, India

**Keywords:** Orthodontic appliances, removable, biofilms, microbiota, bacterial adhesion

## Abstract

Microbial accumulation on clear aligners poses a risk for dental and periodontal health, particularly when proper hygiene practices are not maintained. Therefore, it is of interest to evaluate and compare microbial growth on three different aligner materials: Invisalign, Straumann Clear Correct and Novoalign. An *in vivo* observational study was conducted on 30 subjects, divided into three groups based on the type of aligner used. Microbial samples were collected at baseline (unused aligners, T0) and after 14 days of intraoral use (T1). Samples were cultured using Soybean Casein Digest Agar and Mannitol Salt Agar to quantify *Streptococcus mutans*, *Staphylococcus aureus* and *Lactobacillus* spp. All tested aligner materials exhibited microbial colonisation, with differences based on material composition and surface characteristics. Emphasis on hygiene practices is essential during aligner therapy.

## Background:

Clear aligners have emerged as a popular alternative to traditional fixed orthodontic appliances due to their aesthetic appeal, comfort and removability. These aligners, typically made from thermoplastic materials like polyurethane and polyethylene terephthalate glycol (PETG), allow for better oral hygiene compared to conventional brackets and wires. However, their exposure to the oral environment still renders them susceptible to microbial colonisation [1]. The oral cavity harbours a diverse range of microorganisms, many of which can adhere to aligner surfaces and form biofilms. Although aligners are removable, areas such as attachment dimples and marginal ridges often facilitate bacterial retention. Studies have shown that biofilms on aligners commonly contain *Streptococcus mutans, Staphylococcus aureus and Lactobacillus* species, which are known to contribute to dental caries and periodontal inflammation [2]. Surface characteristics of aligner materials such as roughness, hydrophilicity and surface energy play a crucial role in microbial adhesion. Aligners with smoother and more hydrophilic surfaces tend to exhibit reduced bacterial colonisation. On the other hand, textured or porous surfaces provide retention sites that promote biofilm accumulation [3]. Patient compliance also significantly influences microbial load. Poor oral hygiene practices, extended aligner wear without cleaning and improper storage can lead to increased bacterial adhesion. Bozkurt *et al.* observed that microbial contamination was significantly higher among patients who failed to clean or store their aligners appropriately [4]. In an effort to reduce bacterial colonization, recent studies have explored the use of antimicrobial coatings and nanoparticle-infused materials in aligner design. Although promising, such innovations require further evaluation to assess long-term safety, efficacy and impact on mechanical properties [5]. Therefore, it is interest to 'evaluate and compare the microbial growth associated with three commercially available aligner systems-Invisalign, Straumann Clear Correct and NovoAlign-after 14 days of clinical use.

## Materials and Methods:

This *in vivo* observational study was conducted on 30 orthodontic patients undergoing clear aligner therapy. Participants were divided into three equal groups (n = 10 per group), based on the brand of aligner they were using: Group 1 - Invisalign (Figure 1 - see PDF), Group 2 - Straumann Clear Correct (Figure 2 - see PDF) and Group 3 - NovoAlign (Figure 3 - see PDF). Ethical clearance was obtained from the Institutional Ethical Committee prior to commencement and informed consent was taken from all participants. Unused aligners were collected from each brand (T0) and used aligners were retrieved after 14 days of continuous intraoral wear (T1). Patients were instructed to follow their regular hygiene practices without any modifications. In (Figure 4, 5, 6 - see PDF) Upon removal, each aligner was immediately placed in sterile peptone water as a transport medium and transferred to the microbiology laboratory within one hour of collection to ensure sample integrity. Microbiological analysis involved culturing the samples on Soybean Casein Digest Agar (SCDA) (Figure 7 - see PDF) and Mannitol Salt Agar (MSA) (Figure 8 - see PDF) to isolate and quantify colonies of *Streptococcus mutans, Staphylococcus aureus and Lactobacillus* species. Colony-forming units (CFUs) were calculated after incubation at 37°CC for 24-48 hours under appropriate conditions. Statistical analysis was performed using SPSS software. Paired t-tests were used to compare microbial counts between T0 and T1 within each group. Intergroup comparisons at T1 were carried out using one-way ANOVA, followed by Tukey's post hoc test to determine pairwise significance. A p-value of less than 0.05 was considered statistically significant.

## Results and Discussion:

All three aligner groups demonstrated a measurable increase in microbial load after 14 days of intraoral use. The colony-forming units (CFUs) for Streptococcus mutans, Staphylococcus aureus and Lactobacillus spp. were significantly higher at T1 compared to T0 across all groups. This confirmed that microbial colonization occurs consistently over time, regardless of aligner brand. Table 2 (see PDF) presents, In Group 1 (Invisalign), the microbial load at T1 was significantly greater than at T0, with a statistically significant mean difference (p < 0.05). Table 4 (see PDF) presents, Group 3 (Nova align) showed a similar pattern, with a marked increase in bacterial counts at T1 compared to baseline, also achieving statistical significance (p < 0.05). Table 3 (see PDF) presents, Group 2 (Straumann clear correct) 'exhibited the highest overall microbial accumulation among the three groups. The difference in bacterial load between T0 and T1 in this group was the most pronounced and statistically significant (p < 0.001), indicating that Straumann clear correct may be more susceptible to microbial adhesion due to its material composition or surface characteristics. Table 5 (see PDF) presents, Intergroup comparison using one-way ANOVA revealed significant differences in microbial counts at T1 between the three groups (p < 0.05). Table 6 (see PDF) presents, Post hoc analysis showed that the bacterial load in Group 2 was significantly higher than in Group 1 and Group 3. Group 1 also demonstrated higher counts than Group 1, though the difference was less substantial. These results suggest that while all aligner systems support microbial growth to some extent, the extent of colonization varies by brand, potentially due to differences in material surface roughness, hydrophilicity and wear response. Table 1 (see PDF) presents the descriptive statistics, including mean, standard deviation, minimum and maximum values for microbial counts in each group and their respective controls. Figure 10 (see PDF) presents, the mean microbial load was highest in the Straumann Clear Correct group (2.62), followed by Invisalign (1.56) and Novalign (1.13). Figure 9 (see PDF) presents, the corresponding control group values showed the opposite pattern, with Straumann controls having the highest baseline microbial load (mean = 658), followed by Invisalign (420) and Novalign (234). These findings suggest those Straumann aligners, both at baseline and post-use, exhibited greater microbial retention, possibly due to surface characteristics such as roughness and hydrophilicity.

The paired t-test analysis between Group 1 (Invisalign) and its control group revealed a statistically significant difference in microbial counts (p = 0.000). The mean difference of 418.44 indicates increased microbial colonization on the Invisalign aligners compared to baseline. The 95% confidence interval (407.27 to 429.65) confirms the reliability of the observed increase. This suggests that despite its relatively smoother PETG surface, Invisalign aligners are still susceptible to biofilm formation during usage. A significant increase in microbial count was observed in Group 2 when compared to its control group (p = 0.000), with a mean difference of 107.32. The 95% confidence interval ranged from 641.56 to 669.19. These results indicate that Straumann aligners accumulated the most microbial load during clinical use. The higher colonization may be attributed to the material's surface characteristics that favor bacterial adhesion. Group 3 (Nova Align) also demonstrated a statistically significant increase in microbial count compared to its control (p = 0.000), with a mean difference of 232.87. Despite the significance, the microbial load in Nova Align was the lowest among all experimental groups, suggesting superior resistance to biofilm formation. This could be linked to lower surface energy and smoother texture that inhibit bacterial adhesion. The one-way ANOVA revealed a highly significant difference in microbial counts among the three control groups (p = 0.000, F = 1905.58). This indicates that even at baseline, the material-specific differences in microbial retention were evident. Straumann c ontrols exhibited the highest counts and Nova Align the lowest, reinforcing the influence of material properties on initial microbial adhesion. Post-hoc analysis showed significant differences between all pairs of control groups (p = 0.000). Straumann control had significantly higher microbial count than both Invisalign and Novalign controls. The largest difference was observed between Straumann and Novalign (mean diff = 424), suggesting that Novalign surfaces are less conducive to microbial colonization even without intraoral exposure. These findings underline the material's intrinsic resistance to bacterial adherence. Table 7 (see PDF) presents, The ANOVA test for experimental aligner groups also showed statistically significant differences in microbial counts (p = 0.000, F = 33.065). Straumann had the highest bacterial load, followed by Invisalign and Novalign. These differences are likely attributed to the composition, surface texture and hydrophilic/hydrophobic nature of the materials. Table 8 (see PDF) presents, Pairwise comparisons confirmed significant differences between all experimental aligner groups. Straumann showed significantly higher microbial load compared to Novalign (mean diff = 1.5, p = 0.000) and Invisalign (mean diff = 0.8, p = 0.001). Novalign had significantly lower microbial load than both other groups. Clear aligners are often preferred over fixed appliances due to their removability, comfort and aesthetics. However, this study confirms that despite these advantages, microbial accumulation remains a significant concern. The results demonstrated that all three tested aligner systems exhibited microbial growth after 14 days of wear, reinforcing the findings of Martinez Gil - Ortega *et al.* (2025), who reported that aligner material composition plays a vital role in bacterial adhesion [6]. Straumann Clear Correct exhibited the highest microbial load, while NovoAlign showed the least. This aligns with Moradinezhad *et al.* (2024), who found that increased surface roughness contributes to enhanced microbial retention, making some materials more prone to colonization [7]. In Figure 11, 12 and 13, (see PDF)the presence of *S. mutans, S. aureus and Lactobacillus* in the samples supports previous findings that aligners can act as reservoirs for cariogenic and opportunistic bacteria. Rossini *et al.* (2015) emphasized that material surface characteristics, including transparency and smoothness, influence biofilm development - smoother materials generally resist colonization better than rougher ones [8]. Similarly, Sifakakis *et al.* (2018) compared various aligner brands and found notable differences in bacterial load depending on material properties and wear duration [9]. The relatively lower microbial accumulation on NovoAlign in this study might be attributed to favorable surface characteristics or lower porosity. However, all aligners demonstrated some degree of biofilm formation, highlighting the importance of patient education and maintenance protocols. Monterde *et al.* (2025) concluded that inadequate hygiene during aligner therapy could lead to increased microbial accumulation, reinforcing the need for material-specific hygiene recommendations [10].

## Conclusion:

This *in vivo* study revealed that all three tested aligner systems-Invisalign, Straumann Clear Correct and NovoAlign- exhibited microbial colonization after 14 days of intraoral use. Among them, Straumann Clear Correct showed the highest bacterial accumulation, while NovoAlign demonstrated the least. The findings emphasize the importance of understanding material-specific behavior in microbial retention, incorporating this knowledge into clinical decision-making may enhance oral hygiene outcomes and reduce the risk of microbial complications during clear aligner therapy.

## Clinical significance:

Microbial accumulation on clear aligners poses a risk for dental and periodontal health, particularly when proper hygiene practices are not maintained. The present study demonstrates that different aligner brands exhibit varying levels of microbial adhesion, which may influence treatment safety. Clinicians should consider these differences when selecting aligner systems and emphasize patient education on rigorous hygiene protocols. Further, material-specific cleaning recommendations may help optimize aligner therapy outcomes and reduce biofilm-associated complications.
